# A comparison of microelectrodes for a visual cortical prosthesis using finite element analysis

**DOI:** 10.3389/fneng.2012.00023

**Published:** 2012-09-28

**Authors:** Emma Brunton, Arthur J. Lowery, Ramesh Rajan

**Affiliations:** ^1^Department of Electrical and Computer Systems Engineering, Monash UniversityClayton, VIC, Australia; ^2^Monash Vision Group, Monash UniversityClayton, VIC, Australia; ^3^Department of Physiology, Monash UniversityClayton, VIC, Australia

**Keywords:** finite element modeling, microelectrodes, cortical prosthesis, bionic vision, neural stimulation

## Abstract

Altering the geometry of microelectrodes for use in a cortical neural prosthesis modifies the electric field generated in tissue, thereby affecting electrode efficacy and tissue damage. Commonly, electrodes with an active region located at the tip (“conical” electrodes) are used for stimulation of cortex but there is argument to believe this geometry may not be the best. Here we use finite element analysis to compare the electric fields generated by three types of electrodes, a conical electrode with exposed active tip, an annular electrode with active area located up away from the tip, and a striped annular electrode where the active annular region has bands of insulation interrupting the full active region. The results indicate that the current density on the surface of the conical electrodes can be up to 10 times greater than the current density on the annular electrodes of the same height, which may increase the propensity for tissue damage. However choosing the most efficient electrode geometry in order to reduce power consumption is dependent on the distance of the electrode to the target neurons. If neurons are located within 10 μm of the electrode, then a small conical electrode would be more power efficient. On the other hand if the target neuron is greater than 500 μm away—as happens normally when insertion of an array of electrodes into cortex results in a “kill zone” around each electrode due to insertion damage and inflammatory responses—then a large annular electrode would be more efficient.

## Introduction

Brain machine interfaces, although still in their infancy, already hold much promise in terms of the remediation of brain and sensory deficits (Maynard, 2001; Cogan, 2008). One major area of work in the development of such prosthetic devices is in vision (Rizzo and Wyatt, 1997; Normann et al., 1999; Tehovnik et al., 2005). Two approaches have generally been taken in developing a visual prosthesis. The first is to stimulate the retinal ganglion cells at the back of the eye (Tsai et al., 2009; Zrenner et al., 2009; Humayun et al., 2012). The second approach is to stimulate the visual cortex located at the back of the brain (Brindley and Lewin, 1968; Dobelle and Mladejovsky, 1974; Troyk et al., 2005). In recent developments, stimulating the retina has elicited promising results with groups such as Second Sight successfully developing a prosthesis which is now undergoing clinical trials in the United States and Europe (Humayun et al., 2012). Furthermore Veraart et al. (1998) has developed a cuff electrode in order to stimulate the optic nerve to produce visual sensations. However, retinal and optic nerve prostheses are unable to treat the types of blindness caused by damage to the optic nerve. Cortical visual prostheses have with them the hope that they can treat all types of blindness except for the 7% that affects the brain directly.

The feasibility of producing a visual prosthesis for the blind via electrical stimulation of the visual cortex has been demonstrated in many studies (Brindley and Lewin, 1968; Dobelle and Mladejovsky, 1974; Schmidt et al., 1996) and it is now well established that stimulating the surface of the visual cortex can induce spots of light termed phosphenes (Brindley and Lewin, 1968; Dobelle and Mladejovsky, 1974; Dobelle, 1999). More recently it has been shown that by using electrodes that penetrate into the depth of the visual cortex, more localized regions of tissue can be stimulated, allowing for the perception of more localized phosphenes (Bak et al., 1990; Schmidt et al., 1996). Secondly these studies have shown that intracortical electrodes have greatly reduced thresholds to generate phosphenes, in the range of 0.4–4.6 nC ph^−1^ compared to 200,000 nC ph^−1^ when using surface electrodes (Cogan, 2008). For this reason current visual cortical prosthetic devices, including the one under development at our home institution (Monash University), aim to use penetrating electrodes. The present modeling study was carried out in the context of an attempt to develop a visual cortical prosthesis and the focus is on the geometry of the electrodes for applying electrical signals to the brain.

Designing the geometry of the electrode is important in developing any brain prosthesis as the geometry of the electrode will alter the electric field generated in the tissue (Butson and McIntyre, 2006). This may contribute to tissue damage (McCreery et al., 1990; Shannon, 1992), corrosion of the electrode (Merrill et al., 2005) as may affect the electrode's efficacy (Butson and McIntyre, 2006). The most common penetrating electrode design used for stimulation of the visual cortex has the active area located in the tip (Bak et al., 1990; Campbell et al., 1991; Schmidt et al., 1996; Tehovnik, 1996; Cogan et al., 2004). However, little work has been done to determine whether this geometry is the best for reducing tissue damage and increasing power efficiency.

Finite element computer modeling (FEM) allows the electric fields to be predicted and compared in a highly controlled environment (McIntyre, 2009). McIntyre and Grill (2001) used FEM to model the fields generated by tip microelectrodes and recommended that, to reduce tissue damage, due to an uneven distribution of current density on the surface of the electrode, an electrode with a relatively large surface area, relatively blunt tip and a thin moderately resistive coating should be used. However, a blunt tip makes it more difficult for the electrode to penetrate the thick connective tissue outer meninges layers covering the cortex, and larger tip electrodes require higher threshold currents (Bagshaw and Evans, 1976; West and Wolstencroft, 1983; Tehovnik et al., 2005) and a resistive coating increases the impedance of the electrode resulting in a higher electrode drive power being required. This indicates a need for novel electrode designs to be investigated to minimize damage, without reducing electrode efficacy.

Previously, the electric fields generated by some electrodes with novel geometries have been examined for epidural spinal cord and deep brain stimulation (Grill and Wei, 2009; Wei and Grill, 2009). While differences in brain geometry and electrode size constrain the direct transfer of findings to the design of an electrode for a visual cortical prosthesis, these studies do provide important ideas that can guide consideration of appropriate visual cortical electrodes. These include the idea of segmenting the active area of the electrode, e.g., creating a segmented active area by applying rings of insulation to the active area (Wei and Grill, 2005) or creating an electrode contact with a serpentine perimeter (Grill and Wei, 2009; Wei and Grill, 2009); both create a non-uniform current density on the surface and more efficiently activate neurons located at positions further from the electrode surface. Another option is to move the electrode contact away from the tip, as in deep brain stimulating electrodes (Butson and McIntyre, 2005, 2006; Miocinovic et al., 2009; Yousif and Liu, 2009). Moving the electrode contact away from the tip reduces the risk of damaging the active area of the electrode during insertion. At the same time the surface area of the electrode can be increased, reducing the electrode's impedance, without increasing the activating height, or the tip angle.

Here we aim to compare the electric fields generated by three different electrode geometries (described in detail below in “Geometry of Modeled Electrodes”) that could potentially be used in a visual prosthesis. These electrode designs were chosen as they are easy to fabricate with current manufacturing techniques. While the fields they generate have been examined individually, they have yet to be compared for the purpose of use in a visual cortical prosthetic device.

## Materials and methods

### Geometry of modeled electrodes

**Figure 1:**
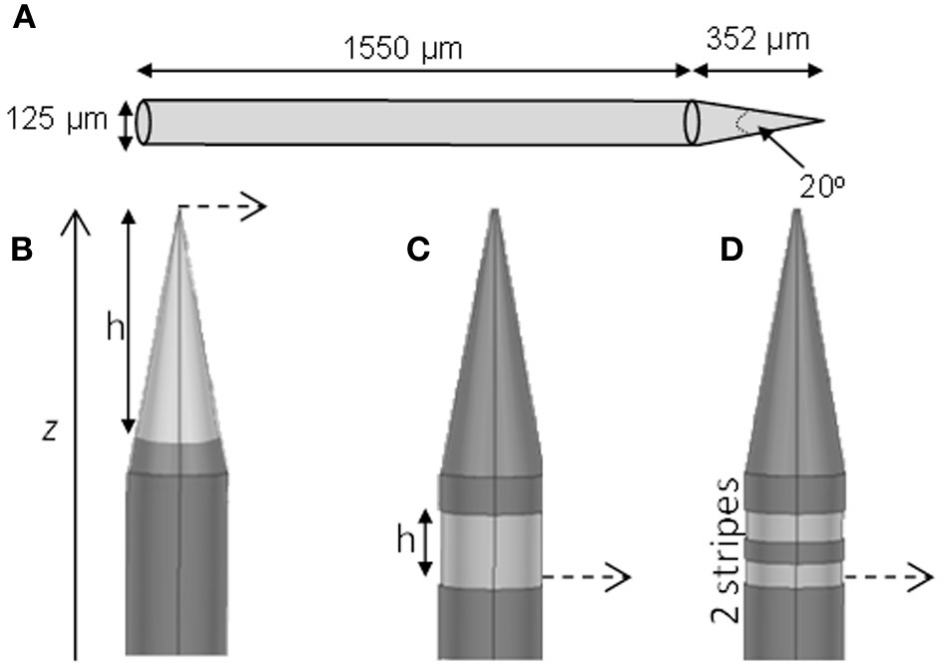
**Electrode geometry**. The geometry of the different electrodes studied. **(A)** Dimensions of the electrode shaft. **(B)** Conical type electrode. **(C)** Annular type electrode. **(D)** Striped electrode. The potential was calculated at points along the dashed line and presented in Section “Voltage Decay Away from the Electrode Surface.” The dark gray represents the insulation and the lighter gray indicates the active regions.

We modeled three types of electrodes, namely the standard tip active electrode (Figure 1B “conical electrode,” where the active area is cylindrical and located away from the tip), an electrode consisting of single active area extending as an annulus across the width of the electrode (Figure 1C “annular electrode”), and an electrode consisting of multiple separated active areas each extending as an annulus across the width of the electrode (Figure 1D, “striped electrode”)—the latter a design suggested by Wei and Grill (2005) to improve the electrode's efficiency.

The size and shape of the electrode shaft was kept constant for all three electrode types and only the area of the electrode covered with insulation was altered, to change the length and geometry of active (non-insulated) segments. Thus the shaft always had a base diameter of 125 μm, and a total length of 1902 μm (Figure 1A). The electrode had a constant length of 1550 μm, and then tapered to a point with an angle of 20°. The end of the tip was blunted so that the tip diameter was 1 μm. The electrode shaft was kept constant, so that differences in geometry would play only a small part in creating damage due to mechanical insertion and this would not need to be considered. The insulation was modeled to have similar properties as Parylene-C (σ = 1.136 × 10^−15^ S/m) and was 3-μm thick. The electrode material was taken to be Platinum/Iridium at a ratio of 80/20 (σ = 3.3 × 10^6^ S/m).

For the conical and annular electrodes we modeled five active-area heights (*h* in Figure 1; for the annular electrode this active area was on the shaft—Figure 1C; for the conical electrode this active area was on the pointed tip region—Figure 1B) of 5, 10, 20, 50, and 100 μm. In addition, a conical electrode with *h* = 125 μm was also modeled. All modeled electrodes are listed in Table 1. For the striped electrodes, annular stripes were spread out over a total height of 100 μm. (i.e., the distance between the bottom of the stripe closest to the base and the top of the stripe closest to the tip), with a combined active segment length of 72 μm. Electrodes with 2, 4, and 8 stripes were modeled; the segments were all electrically continuous under the insulation. For a cortical visual prosthesis we want to target layer 4Cb of the visual cortex (Normann, 1990), thus the height of the electrode is important so that we are not stimulating multiple cortical layers. An electrode with a smaller height would be preferred so that it is more specific in which layers are activated and hence in this study we have placed more emphasis on the height of the electrode rather than the geometrical surface area.

**Table 1:** **List of all electrodes modeled, their geometry, maximum current density and resistance**.

**Electrode type**	**Number of segments**	**Active height (μm)**	**Total height (μm)**	**Surface area (μm^2^)**	**Maximum current density (A/m^2^)**	**Tissue resistance (kΩ)**
Conical	1	5	5	30	874299	602.0
	1	10	10	89	421560	330.0
	1	20	20	289	193253	174.0
	1	50	50	1566	65876	71.4
	1	100	100	5945	28770	36.0
	1	125	125	9189	23623	28.4
Annulus	1	5	5	1964	7681	49.8
	1	10	10	3927	4503	37.2
	1	20	20	7854	2770	28.4
	1	50	50	19635	1513	19.8
	1	100	100	39270	976	14.6
Stripes	2	72	100	28274	669	15.2
	4	72	100	28274	662	15.1
	8	72	100	28274	691	15.0

The maximum current density and resistance were calculated using COMSOL Multiphysics.

### Finite element models

Axis-symmetric finite element models of the electrodes surrounded by an isotropic homogeneous medium representative of brain tissue, σ = 0.1 S/m (Gabriel et al., 1996), were developed in COMSOL Multiphysics (Version 4.0a, Comsol Inc., Stockholm, Sweden). Brain tissue was modeled as a cylinder with radius and height of 20 cm with the outer boundaries that were not touching the electrode set to ground (*V* = 0). This dimension was chosen so that the ground was located sufficiently far away from the electrode so that its location did not interfere with the results of the field generated from each of the electrodes. The models were partitioned into mesh elements using a fine triangular mesh. The models were created so that the same mesh was used for each electrode; the material properties of the individual elements were altered to change the insulated/non-insulating areas of the electrode geometry. This ensured the validity of comparisons made between different electrode geometries. Models were created in 2-D in order to reduce computational cost, however they are representative of a 3-D situation.

The electrode contacts were set to deliver a DC cathodic current of 25 μA (unless otherwise stated), which is close to the upper threshold for the perception of phosphenes in the human visual cortex (Schmidt et al., 1996) and similar to the threshold for activation of motor outputs with stimulation in motor cortex (Tandon et al., 2008). While the frequencies that have been used to generate phosphenes in the visual cortex range from 75 to 200 Hz (Schmidt et al., 1996) a DC current as used in this study is suitable when comparing fields across the tissue, which is resistive. This also reduces the dimensionality of the comparison by removing the capacitance of the electrode from the results. The voltages (Φ) at the nodes of the mesh elements were calculated by solving Laplace's equation ∇^2^ Φ = 0. The current density (*J*) along the surface of the electrode within the mesh elements was calculated using Ohm's law *J* = −σ∇Φ. The maximum current density was calculated as the average current density over the 1% of the electrode surface with the greatest current density. For simplicity we modeled the brain material and ignored the surface effects of the electrode. If we consider that the electrode impedance is connected in series with the tissue resistance, then the current through both elements would be equal. The drive voltage will depend on the electrodes' capacitance and on the stimulation duration, and these have not been considered. There will also be additional currents due to parasitic capacitance of the insulated part of the electrode which also has not been considered. Tissue resistance was calculated using *V* = *IR*, where *I* was the current supplied to the electrode and *V* was the resultant voltage at the electrode surface. The capacitance due to the polarization of the electrode interface was not considered; this would roughly scale with the inverse of the surface area of the electrode (Schwan, 1968).

### Activating function distributions

Changing the field generated in the tissue may alter the pattern of neural excitation. Neural excitation by extracellular sources can be predicted by the activating function (Rattay, 1986) which is proportional to the second spatial derivative of the extracellular potential *f* ∝ ∂^2^*V*/∂*x*^2^. A positive value of the activating functions means that neurons in this region can be stimulated and the higher the value of *f*, the more likely it is that a neuron in this region would be activated (Rattay, 1986). A negative value of *f*, indicates that a neuron in this region will not be activated with the supplied pulse, but would be activated with a pulse of the opposite sign.

**Figure 2:**
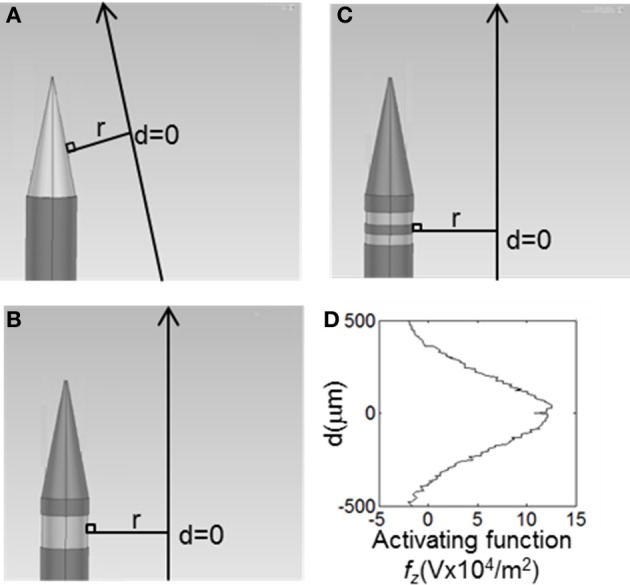
**The value of the activating function in the, z, direction were was calculated at points along the long solid lines, parallel to the electrode surface**. These lines lie, *r*, away from the active surface. The distributions were centred around a line perpendicular from the middle of the active segment on the electrode, shown as *d* = 0. **(A)** Conical electrodes, **(B)** Annular electrodes, **(C)** Striped electrodes. **(D)** Value of the activation function for the 100-μm annular electrode at *r* = 500 μm when supplied with 25 μA, as calculated along the line parallel to the electrode surface shown in **(B)**.

Distributions of *f*_*z*_ = ∂^2^*V*/∂z^2^ (for activation of axons oriented parallel to the electrode surface—see Figure 2) were calculated in the tissue surrounding the electrode surface. The value of *f*_*z*_ was calculated along a line parallel to the electrode surface at distances (*r*) of 10, 60, and 500 μm. These positions were chosen to represent the near (*r* = 10 μm), mid (*r* = 60 μm) and far (*r* = 500 μm) fields. The step size, Δ*z*, was equivalent to the mesh spacing, which ranged from ~1 μm at *r* = 10 to ~100 μm at *r* = 500 μm. The activating function distributions were calculated for three cases: a current-controlled mode with the electrode drive set to −25 μA; a voltage-controlled mode where the electrode drive was set to be −1 V; and a power-controlled mode where the power dissipated in the tissue was 1 μW (*P* = IV). The peak value and the full width at half maximum (FWHM) of the activating function distribution, *f*_*z*_, was calculated in order to easily compare the different electrode types. An example distribution is shown in Figure 2D. Thus a higher peak value of *f*_*z*_ indicates a lower current would be required to stimulate neurons. A higher FWHM indicates a larger region of tissue would be stimulated.

## Results

To characterize and compare the different electrode types, four features of the electrode and the field generated by the electrode were used: current density distribution, tissue resistance, the pattern of voltage decay away from the electrode surface, and the activating function, *f*, in the surrounding tissue.

### Current density distribution

**Figure 3:**
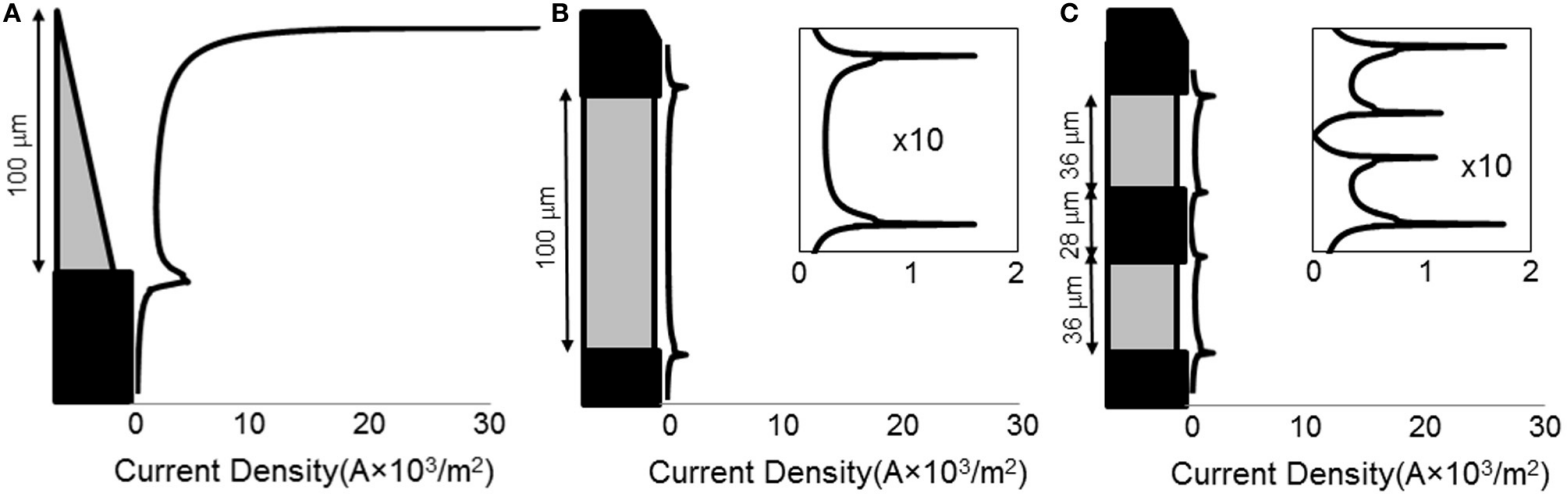
**Line plots of primary current density (Grabowski and Newman, 1993) normal to the surface of the electrode**. Current density as calculated for three different electrode types: **(A)** 100-μm conical; **(B)** 100-μm annulus; **(C)** electrode with two active area stripes. All current densities are shown on the same horizontal scale. For the annular and striped electrodes, the boxed inset shows a zoomed-in view of the current density functions in order to clearly see the shape of the distribution.

When a current of 25 μA was applied, all electrode types show non-uniform current density over the active surface (Figure 3), with the conical electrodes showing the largest difference between maximum current density and average current density. All electrodes show edge effects with local peaks in current density at the electrode-insulation boundaries. The maximum current density occurs at the tip for the conical electrodes, at the electrode/insulation boundaries for the annular electrodes, and at the outermost electrode/insulation boundaries for the striped electrodes. The maximum current densities for the conical electrodes are more than 10 times larger than for the annular and striped electrodes of the same active height (Table 1). Increasing the number of striped segments on the electrode surface reduces the maximum current density for the same active height; however, the solid 100-μm annulus, which has total height equal to that of the striped electrodes, has the lowest maximum current density when supplied with the same current.

### Tissue resistance

**Figure 4:**
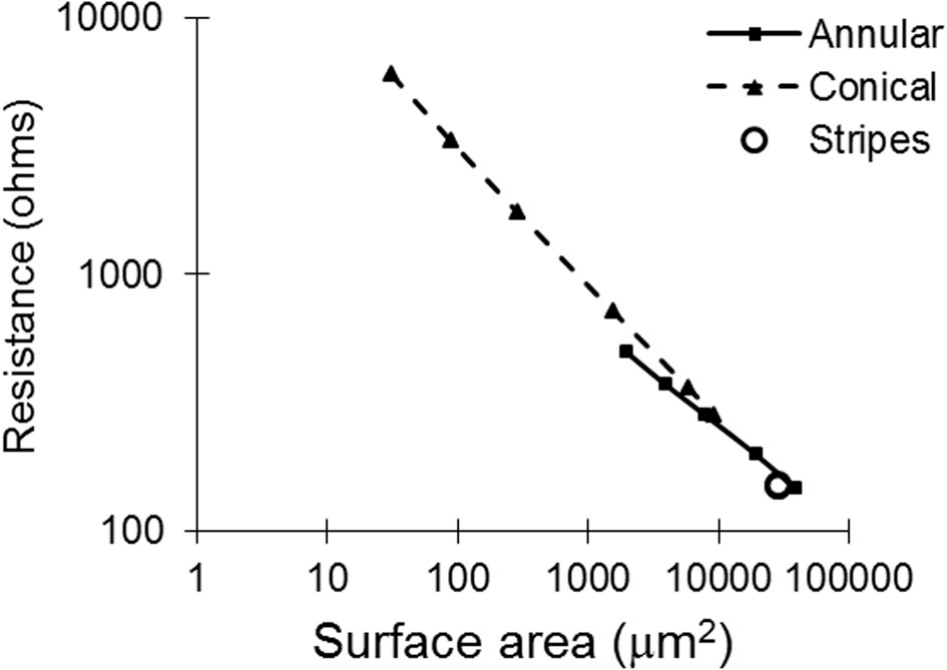
**Tissue resistance plotted against surface area for all of the electrode types, as calculated with COMSOL**.

With regard to the resistances of the different electrode types (calculated by COMSOL; Figure 4), as expected, as the surface area is increased, the resistance of the electrode decreases. The electrode with striped insulation had low resistance and increasing the number of stripes reduced the resistance even more (Table 1). However, overall, the lowest resistance was found with the solid 100-μm annular electrode which has the same total height as the electrodes striped with insulation. This is as expected as this electrode has the greatest exposed surface area.

### Voltage decay away from the electrode surface

**Figure 5:**
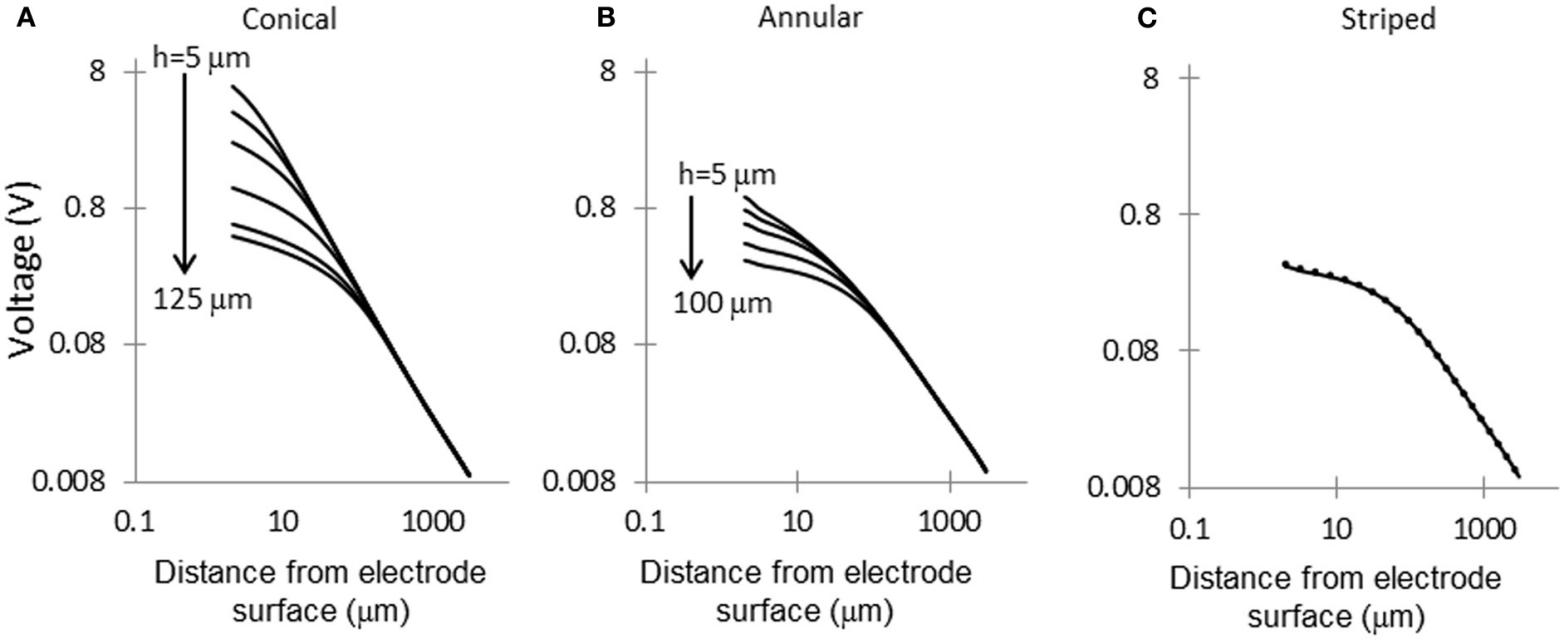
**Voltage decay in tissue, away from electrode surface**. Voltage decay was calculated along the dashed line shown in Figure 1. **(A)** Conical electrodes: 5, 10, 20, 50, 100, and 125 μm; **(B)** Annular electrodes: 5, 10, 20, 50, and 100-μm; **(C)** Striped electrodes: 2, 4, and 8 stripes.

Figure 5 shows the electric potential calculated at points along the dashed line shown in Figure 1. For small area electrodes the field is very high close to the exposed part of the electrode, as expected from the Laplace equation. Away from the electrodes the field becomes independent of the configuration of the source. The electrodes striped with insulation follow a similar pattern of voltage decay as the 100-μm annulus.

### Activating function distributions

Profiles of the discrete second spatial derivatives, *f*_*z*_ = Δ^2^*V*_*e*_/Δ*z*^2^, were plotted along a parallel line, at three electrode-to-axon distances, *r*, for three different electrode supply conditions; electrodes supplied with 25 μA, electrodes supplied with 1 V, and electrodes supplied with 1 μW. From these distributions the maximum value of *f*_*z*_ and the value of the FWHM was obtained.

#### Current-controlled mode: electrodes supplied with 25μA

**Figure 6:**
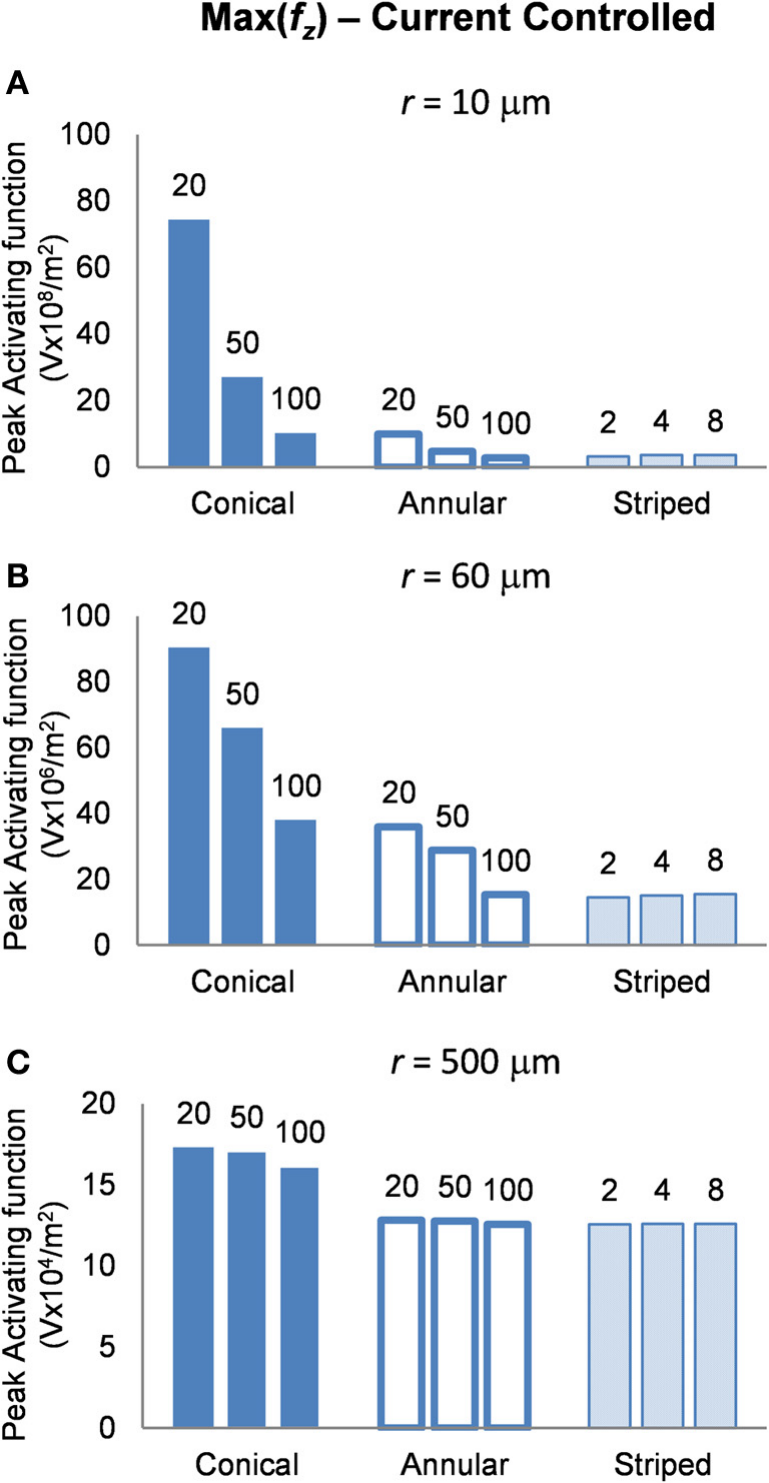
**Maximum values of the activating function as calculated along a line parallel to the electrode surface, shown in Figure 2, when the electrode is supplied with −25 μA at distances of: (A) *r* = 10 μm; (B) *r* = 60 μm; (C) *r* = 500 μm**. Note change in y-axis between **(A)**, **(B)**, and **(C)**.

In interpreting the results from the current-controlled mode, it must be borne in mind that higher values of the activating function are better as they indicate that a lower current is required to stimulate neurons located at the given distance away from the electrode surface. From Figures 6A–C it can be seen that at all distances from the electrode surface that were measured, the conical electrodes (with the smallest surface area) generated higher maximum values of *f*_*z*_ than either the annular or the striped electrodes. Striping the electrode surface with insulation results in similar maximum values of the activating function to the 100-μm annulus electrode. Detailed results can be found in Table 2.

**Table 2:** **Current controlled mode, peak activating function at distances *r* = 10, 60, and 500 μm**.

**Electrode**		**Peak activating function**		
		***r* = 10 (V × 10^8^/m^2^)**	***r* = 60 (V × 10^6^/m^2^)**	***r* = 500 (V × 10^4^/m^2^)**
Conical	20 μm	74.39	90.47	17.31
	50 μm	27.08	66.01	17.08
	100 μm	10.23	37.96	16.14
Annulus	20 μm	9.92	35.96	12.81
	50 μm	4.76	28.74	12.77
	100 μm	2.82	15.34	12.57
Striped	2 stripes	3.16	14.49	12.54
	4 stripes	3.48	15.02	12.57
	8 stripes	3.54	15.45	12.58

The highest peak activating function has been highlighted for each distance.

#### Voltage-controlled mode: electrodes supplied with 1 V

**Figure 7:**
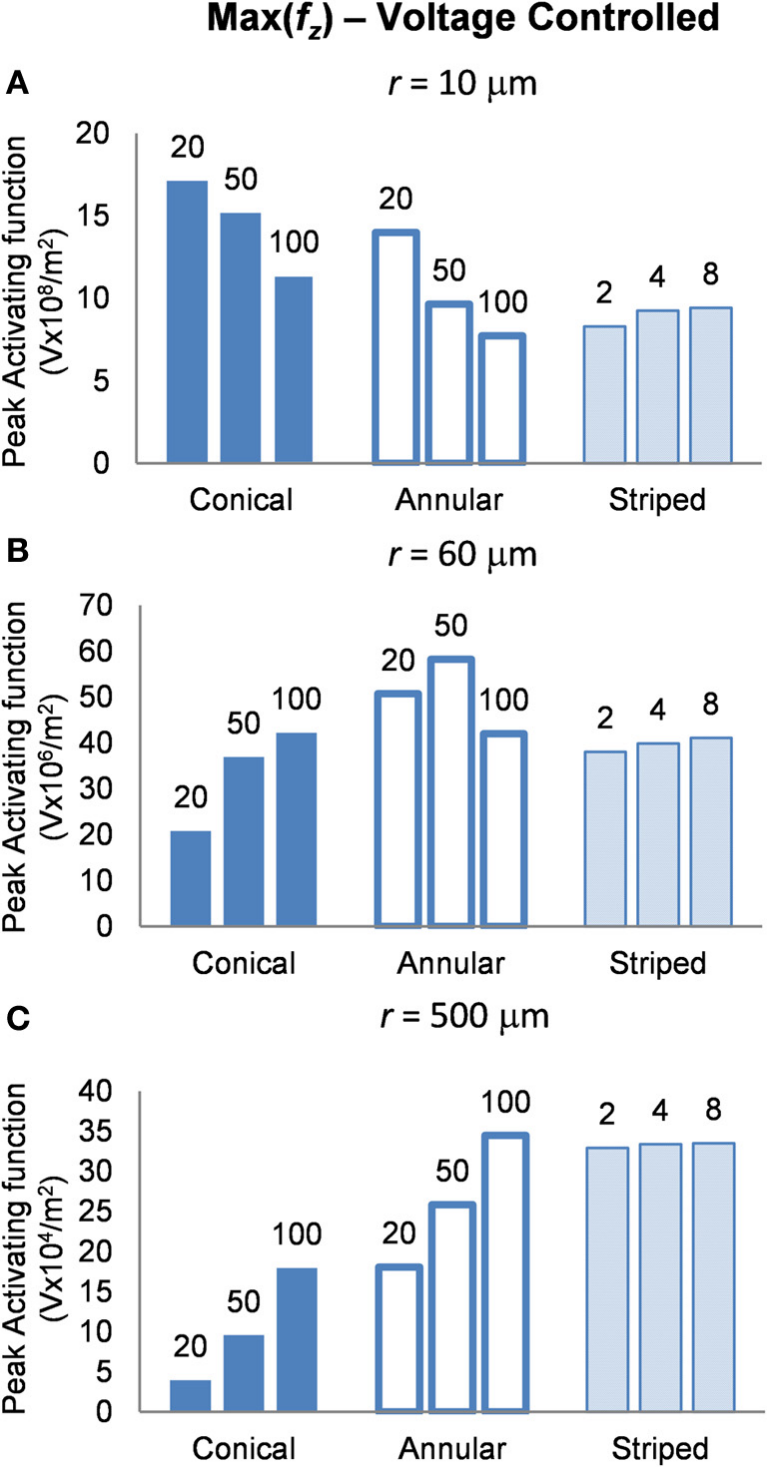
**Maximum values of the activating function as calculated along a line parallel to the electrode surface, shown in Figure 2, when the electrode is supplied with −1 V at distances of: (A) *r* = 10 μm; (B) *r* = 60 μm; (C) *r* = 500 μm**. Note change in y-axis between **(A)**, **(B)**, and **(C)**.

In this mode, as in the current-controlled mode, higher values of the activating function are better as they indicate that a lower voltage is required to stimulate neurons located at the given distance away from the electrode surface. When the electrode surface was set to be 1 V (Figure 7) the conical electrodes (with the smallest surface area) had a higher maximum value of *f*_*z*_ in their near field only (Figure 7A). At *r* = 60 μm the 50-μm annulus had the largest maximum value of *f*_*z*_ (Figure 7B). At *r* = 500 μm the 100-μm annulus had the largest maximum value of *f*_*z*_ (Figure 7C). Detailed results can be found in Table 3.

**Table 3:** **Voltage controlled mode, peak activating function at distances *r* = 10, 60, and 500 μm**.

**Electrode**		**Peak activating function**		
		***r* = 10 (V × 10^8^/m^2^)**	***r* = 60 (V × 10^6^/m^2^)**	***r* = 500 (V × 10^4^/m^2^)**
Conical	20 μm	17.11	20.80	3.98
	50 μm	15.16	36.96	9.56
	100 μm	11.38	42.21	17.94
Annulus	20 μm	13.98	50.67	18.05
	50 μm	9.63	58.20	25.85
	100 μm	7.72	42.02	34.44
Striped	2 stripes	8.29	38.02	32.90
	4 stripes	9.24	39.86	33.38
	8 stripes	9.42	41.12	33.50

The highest peak activating function has been highlighted for each distance.

#### Power-controlled mode: electrodes supplied with 1 μW

**Figure 8:**
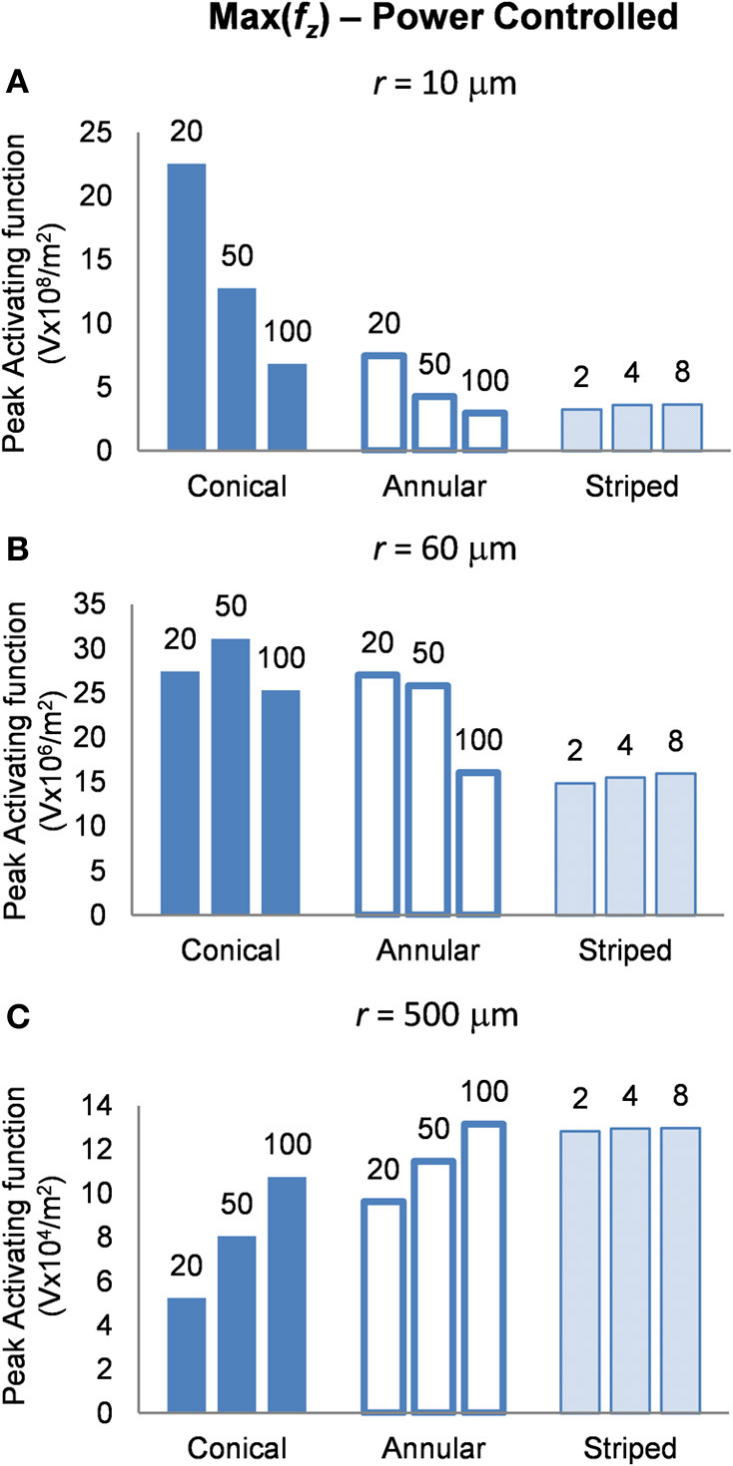
**Maximum values of the activating function as calculated along a line parallel to the electrode surface, shown in Figure 2, when the electrode is supplied with 1 μW at distances (A) *r* = 10 μm (B) *r* = 60 μm and (C) *r* = 500 μm**. Note change in y-axis between **(A)**, **(B)**, and **(C)**.

Finally, in this mode, a higher peak value of *f*_*z*_ is better as it indicates less power will be required in order to stimulate neurons at the given distance. Figure 8 shows the activating function distributions when the electrode contacts were set to deliver 1 μW. In the near field the 20-μm conical electrode had the largest maximum value of *f*_*z*_ (Figure 8A). By comparison, in the mid field the 50-μm conical electrode had the largest maximum value of *f*_*z*_ (Figure 8B). Finally, in the far field the electrode with the largest surface area, the 100-μm annulus electrode had the largest maximum value of *f*_*z*_ (Figure 8C). Detailed results can be found in Table 4.

**Table 4:** **Power controlled mode, peak activating function at distances *r* = 10, 60, and 500 μm**.

**Electrode**		**Peak activating function**		
		***r* = 10 (V × 10^8^/m^2^)**	***r* = 60 (V × 10^6^/m^2^)**	***r* = 500 (V × 10^4^/m^2^)**
Conical	20 μm	22.53	27.40	5.24
	50 μm	12.76	31.12	8.05
	100 μm	6.82	25.31	10.76
Annulus	20 μm	7.46	27.02	9.62
	50 μm	4.28	25.83	11.47
	100 μm	2.95	16.06	13.16
Striped	2 stripes	3.24	14.84	12.84
	4 stripes	3.59	15.47	12.96
	8 stripes	3.65	15.94	12.99

The highest peak activating function has been highlighted for each distance.

#### Volume of tissue activated

**Figure 9:**
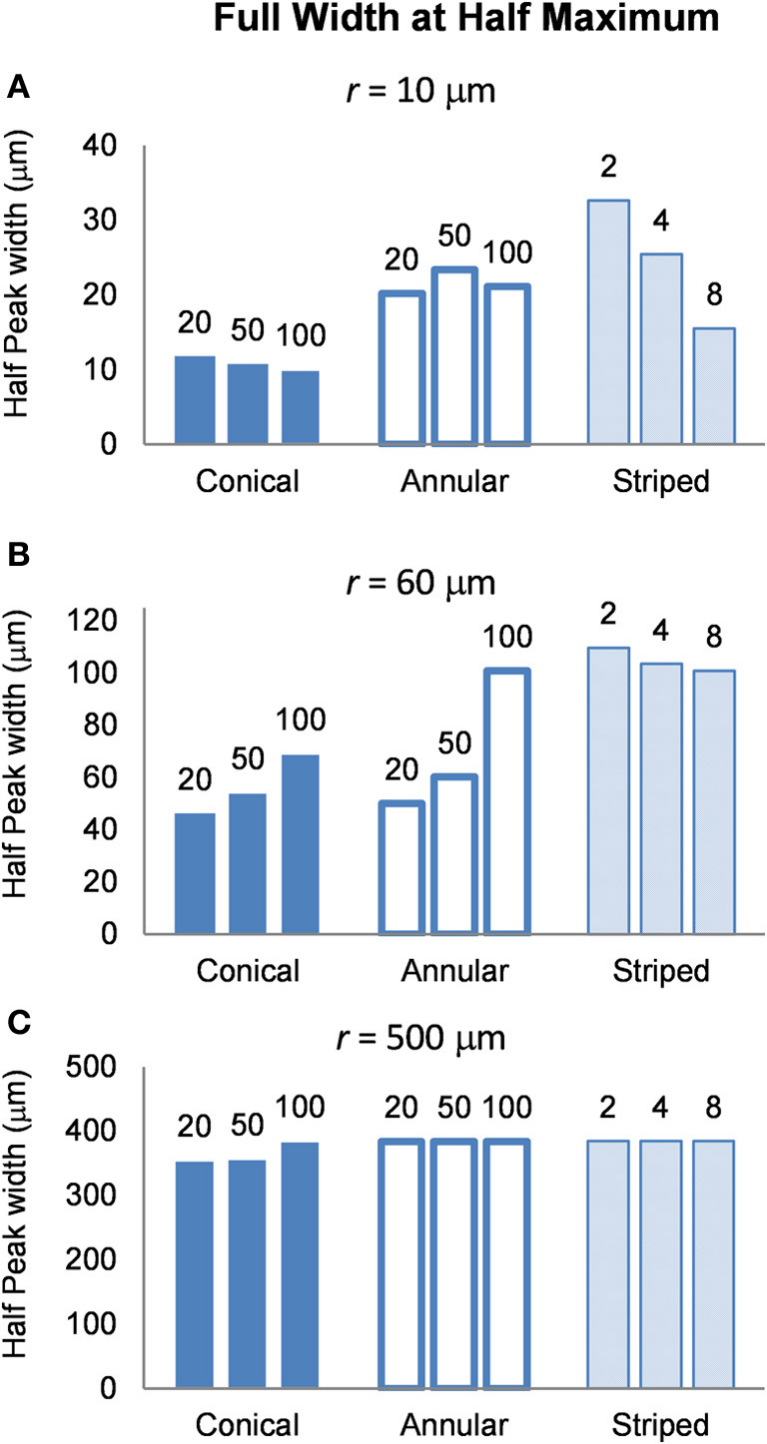
**FWHM of the distributions of the activating function, calculated at distances (A) *r* = 10 μm (B) *r* = 60 μm and (C) *r* = 500 μm**. The shape of the distribution is independent of the drive condition and so the FWHM will be the same for current- voltage- and power controlled modes.

To determine the area of tissue activated, we calculated the FWHM of *f*_*z*_ for the different electrode types (Figure 9). Note that a higher FWHM indicates that a larger volume of tissue is stimulated at or just above threshold. As the shape of the electric field generated in the tissue does not depend on the drive condition, the FWHM will be the same for all drive conditions. Figure 9 shows that the electrode with two stripes has the highest value of the FWHM at all distances examined in this study. However, in the far field the FWHM of all electrode types is similar (Figure 9C). Detailed results can be found in Table 5.

**Table 5:** **FWHM of the activating function at distances *r* = 10, 60, and 500 μm**.

**Electrode**		**FWHM (μm)**		
		***r* = 10**	***r* = 60**	***r* = 500**
Conical	20 μm	11.80	46.24	354.58
	50 μm	10.78	54.27	357.06
	100 μm	10.01	69.16	384.58
Annulus	20 μm	20.19	50.00	383.92
	50 μm	23.38	60.17	383.92
	100 μm	21.13	100.87	383.92
Striped	2 stripes	32.60	109.65	383.92
	4 stripes	25.41	103.46	383.92
	8 stripes	15.52	100.87	383.92

The largest FWHM has been highlighted for each distance.

## Discussion

The objective of this modeling study was to compare three different electrode geometries that could be used for a cortical visual prosthesis. When choosing an appropriate electrode design for a visual prosthesis, one needs to consider the damage to the tissue as well as stimulation efficacy.

### Tissue damage

With neural prostheses there are a number of factors that affect neural tissue damage but, with respect to electrical stimulation, the charge density and charge per phase have been found to be important cofactors in causing neural damage (Agnew et al., 1983). Many studies have shown that increased charge density at the tip of the electrode is associated with increased histological damage (Pollen, 1977; Yuen et al., 1981; Agnew et al., 1983; McCreery et al., 1990). Charge density is the product of current density and pulse duration. Shannon (1992) used the results collected by McCreery to develop an expression for the maximum safe level of stimulation which is $\log\left(\frac{Q}{A}\right)=k-\text{log}(Q)$ where *Q* is the charge per phase (μC/ph) and, *A*, is the surface area of the electrode (cm^2^). From McCreery's data on the cat cerebral cortex Shannon determined a safe, *k*, value of 1.5 over a charge density range of 1–1000 μC/cm^2^. Reducing the charge density on the surface of the electrode allows for a greater charge per phase to be used before histological damage is seen.

Results of this study show that electrode size and geometry greatly affects the current density distribution on the surface. These results show that the annular electrodes distribute the current more evenly on the surface than the conical electrodes; this could reduce the likelihood for tissue damage. The maximum current density on the surface of a conical electrode with the same height as an annular electrode can be up to 10 times greater due to its smaller surface area. If electrode height is kept constant in order to target a specific cortical layer, then the annular electrode would be able to provide a greater charge per phase to the tissue before histological damage would be seen. However, histological studies will need to be performed to determine whether the maximum current density on the surface of the electrode is correlated with levels of tissue damage and these are planned in the future.

In order to reduce tissue damage, the development of electrodes with a highly uniform current density distribution on their surface has been investigated. These studies have shown that by recessing the electrode, the surface current density can be made almost uniform (Rubinstein et al., 1987; Ksienski, 1992). In this study, the annular electrodes were only recessed from the surface of the insulation by 3 μm, i.e., the insulation was 3-μm thick, thus the distance between the surface of the metal electrode and the surface of the insulating coating was 3 μm. Potentially, the current density of the annular electrodes could be made more uniform by increasing the thickness of the Parylene coating, further reducing the propensity for tissue damage.

### Neural excitation

The pattern of neural excitation was determined by calculating values of the activating function generated in the tissue for three conditions: current controlled stimulation, voltage controlled stimulation and power controlled stimulation.

#### Current controlled

The conical electrodes have a higher maximum value of *f*_*z*_ at all distances, suggesting that they need a lower current to activate an axon at any distance when compared with the annular or striped electrodes. Figure 6A indicates that electrodes with a smaller active height produce higher maximum values of *f*_*z*_ in the near field. If we assume that, at the threshold for activating a cortical area, activation occurs only for neurons in the near field of an electrode (i.e., *r* = 10 μm), then we would expect to see that electrodes with smaller active heights would require lower currents at threshold. This concurs with experimental work by Bagshaw and Evans (1976) who found that more current was required to stimulate the sciatic nerve of a frog when using large-tipped electrodes than small-tipped electrodes. Similarly, West and Wolstencroft (1983) found that to evoke action potentials from reticulospinal neurons, much higher current was needed with large-tipped electrodes than with small-tipped electrodes.

In the far field, the configuration of the source becomes negligible and the fields begin to look similar. This means that to stimulate axons distant from the electrode (i.e., in the far field), electrode height and geometry will have little effect. Then, if we assume that saturation of electrically-activated neural effects is due to current spread to stimulate the far field, the current level where saturation occurs should remain similar for all electrode types. This implies that for electrodes with a larger active height, the difference between threshold current and saturation current would be reduced.

#### Voltage controlled

It is clear from Figure 7, that in order to select an electrode that reduces the voltage required to stimulate a given axon, the distance of the axon to the electrode needs to be carefully considered. In order to stimulate an axon in the near field, the 5-μm conical electrode requires the lowest voltage. In the mid field the 50-μm annulus electrode requires the lowest voltage. In the far field the 100-μm and the striped annulus electrodes require the lowest voltage. As shown in Figure 5, because the total current is passing through a smaller volume, the majority of the voltage drop in the bulk tissue occurs in the region of tissue closest to the electrode surface.

#### Power consumption

Altering the electrode's geometry has previously been demonstrated as a way to reduce power consumption of an implanted electrical prosthesis (Grill and Wei, 2009; Wei and Grill, 2009). Traditionally, it has been viewed that to make the electrode more effective, the electrode's resistance should be reduced (McIntyre and Grill, 2001; Wei and Grill, 2005). However, our study suggests otherwise. While an electrode with reduced resistance requires a lower voltage to generate the same current in the tissue, the electrode geometry also affects how this current is distributed in the tissue and thus the ability of the electrodes to activate neurons. Figure 4 illustrates that the electrode with the lowest resistance is the one with a 100-μm annulus. However, when this electrode annulus was supplied with the same power as other electrodes (Figure 8), the maximum value of *f*_*z*_ is one of the lowest, in both near and mid fields. These results suggest that when considering electrode geometry to reduce power consumption, the distance of target neurons from the electrode needs to be considered. For the mid and near fields, a small conical electrode would have the greatest efficiency but in the far field, a large annular or striped electrode would be more efficient.

For a cortical visual prosthesis, if we were to consider a “pure” condition that excludes any damage caused by insertion of the prosthesis, it is likely that target axons will be located close to the electrode surface, i.e., within 10 μm, due to the fact that neuronal density in the visual cortex is approximately 40,000 neurons/mm^3^ (Leuba and Garey, 1989). However, a more realistic scenario should consider that mechanical damage during insertion of the electrode greatly reduces the density of neurons within the near field (McCreery et al., 2010) and that neurons will be further separated from the electrode upon formation of a glial scar as part of the inflammatory response to a foreign body in the cortex (Xindong et al., 1999; Polikov et al., 2005). Studies have reported “kill zones,” where the density of neurons has been significantly reduced, to be anywhere from less than 1 μm to more than 100 μm, with a value of ~100 μm considered to be common (Stensaas and Stensaas, 1976; Turner et al., 1999; Polikov et al., 2005). So while the 5-μm conical electrode has a much higher value of the activating function in the near field for both current- and voltage-controlled stimulation, the density of viable neurons in this region will be greatly reduced and neurons may even be absent in this zone. Schmidt et al. (1996) found that the threshold current for the perception of phosphenes increased from 15.9 μA on day 35 to 24.8 μA on day 78, before stabilizing, when a tip electrode was used. This could be due to glial scar formation, increasing electrode to axon distances. This would alter the optimal distance to target for electrical stimulation and the optimal geometry which should be chosen for stimulation. The geometry of the electrode has the largest effect on the pattern of neural activation in the near field. As the biocompatibility of electrode materials improves, electrode geometry could play a major role in reducing the power consumption of implanted devices.

#### Region of tissue activated

FWHM was used to index the region of tissue that would be activated for each electrode type because it allows for comparison between electrodes and is computationally less costly than other methods that have been proposed to determine the volume of tissue activated. Other methods have relied on coupling multi-compartmental neuron models to electric field data (McNeal, 1976) or expressing activating function threshold as a function of voltage × pulse width for voltage controlled sources (Butson and McIntyre, 2006). It must be noted that although our method is computationally less demanding, it is also less accurate; nevertheless, it serves usefully to give an indication of the volume of tissue that would be activated when the different electrode types are used. A larger FWHM indicates a larger region of tissue is stimulated. The peak of the activating function is much broader for electrodes with larger activating heights (Figure 9), and even broader for electrodes striped with insulation. This implies that a larger volume of tissue and therefore, a greater number of neurons will be activated at threshold, i.e., the electrode will be less specific. The electrode with two stripes of insulation has a FWHM of 32.6 μm which is almost three times that of the 20 μm conical which has a FWHM value of 11.8 μm in the near field. A conical electrode would also activate a smaller region even with the same FWHM of an annular electrode, as the FWHM corresponds to the slant height of a cone, whereas the annulus corresponds to the height of a cylinder.

For the purpose of a visual prosthesis, an electrode that stimulates a large region of tissue may result in more than one phosphene being perceived, comparable to what has been seen by Brindley and Lewin (1968), Dobelle and Mladejovsky (1974), and Dobelle (1999), and this would greatly reduce the resolution of the device. On the other hand, stimulating a larger area of tissue increases the likelihood of a neuron being located in a stimulated region, closer to the electrode's surface. This scenario is especially important when considering that many neurons will be damaged or killed due to insertion of the electrode into the cortex. Increasing the volume of tissue activated will increase the likelihood of a neuron being located close to the electrode surface within the activated zone. As a higher current will be required to activate neurons that are located further away, or that are located in a region of tissue where the activating function is below the half peak value. It must also be noted that the area stimulated by the annular electrodes would be significantly different for the conical electrodes. The conical electrode would primarily stimulate neurons at its tip, downwards from the position of insertion, compared to the annular electrode which would primarily stimulate tissue in a ring to the sides of the electrode. The annular electrode would need to penetrate deeper in to the brain in order for the active region to be located in the same layer as the tip electrode.

### Other considerations

The annulus design has been proposed because on insertion of the device, the active part of the electrode is away from the tip. The tip is the most fragile part of the electrode during insertion and so if the tip is damaged for the annular type electrodes the active area will remain unscathed. However, this also means that the tip of the electrode needs to extend further into the brain in order for the active part of the electrode to sit at the correct depth.

### Model limitations

The electric fields calculated in these finite element models were based on the idealized assumption that the brain is an isotropic, homogeneous material, which is not the case (Miocinovic et al., 2009). Further, this is an electrostatic model of the field, so no frequency effects were accounted for and current distribution on the surface is assumed to be unaffected by the capacitive layer that forms between the electrode surfaces and surrounding tissue. Within these limits, this modeling study aimed to make a simple comparison between different electrode geometries, and therefore the simplified model should suffice. However, due to this simplification the power consumption will be significantly under estimated as we have not accounted for the power dissipated charging the capacitance of the tissue interface. The electrode interface impedance may be significantly higher than the tissue resistance, and this is greatly dependent on the electrode surface area (Schwan, 1968).

Neural excitation was measured by calculating values of the activating function for axons parallel to the surface of the electrode. We acknowledge that the distributions of neurons and axons in the cortex are much more complex, and so calculating the value of the activating function may not accurately reflect neuronal excitation *in situ*. Also if the distributions of the activating function were calculated along different lines, then the electrode most suitable for stimulation may be different. For example, for conical electrodes, the distance from the electrode tip to the neurons may be a better metric, as the tip is more likely to stimulate neurons.

## Conclusions

Electrode geometry is an important consideration when developing a cortical visual prosthesis. Choosing the most efficient electrode geometry is dependent on the distance that the target neurons are from the electrode surface, as well as whether it is preferred to reduce voltage, current, or power. For a visual prosthesis it is likely that neurons around the electrode will be damaged due to insertion of the device. Neurons that do survive the initial insertion will be further separated from the device by formation of a glial scar around the electrode. This “kill zone” may extend further than 100 μm (Polikov et al., 2005). As annular electrodes perform similarly to, if not better than, conical electrodes in terms of power consumption in the mid and far field, and it is likely that the annular configuration of the electrode would reduce the propensity for tissue damage, it is recommended that an annular electrode would be the best suited for a visual prosthesis. While striping the electrode with insulation does not reduce the current required to activate neurons at a certain distance, it may improve the chances of a neuron being located within a stimulated region of tissue, as the FWHM of the activating function is increased.

### Conflict of interest statement

The authors declare that the research was conducted in the absence of any commercial or financial relationships that could be construed as a potential conflict of interest.
